# Attention Deficit Hyperactivity Disorder and other neurodevelopmental traits are associated with impact on functioning among children in the general population

**DOI:** 10.1002/jcv2.70004

**Published:** 2025-03-07

**Authors:** Louise Horstmann, Charlotte A. Dennison, Evie Stergiakouli, Kate Langley, Joanna Martin

**Affiliations:** ^1^ School of Psychology Cardiff University Cardiff UK; ^2^ Wolfson Centre for Young People's Mental Health Cardiff University Cardiff UK; ^3^ Centre for Neuropsychiatric Genetics and Genomics Cardiff University Cardiff UK; ^4^ Population Health Sciences Bristol Medical School University of Bristol Bristol UK

**Keywords:** ADHD, ALSPAC, impact on functioning, neurodevelopmental traits, polygenic score, sex differences

## Abstract

**Background:**

Attention Deficit Hyperactivity Disorder (ADHD) is commonly defined as a categorical diagnosis requiring clinically severe symptoms and impact on functioning. However, ADHD and other neurodevelopmental traits are also distributed continuously in the general population, where their impact on functioning is less clear. This study aimed to examine the association between ADHD impact and (a) ADHD traits, (b) co‐occurring neurodevelopmental traits (autistic traits, reading ability, IQ, and pragmatic communication), and (c) genetic risk for ADHD. We also examined sex differences in these associations.

**Methods:**

We identified 12,439 children with parent or teacher reports of ADHD at ages 8 and 11 in a UK birth cohort. We examined ADHD impact (i.e., in school, home, friendships, leisure activities, and distress) as an outcome of ADHD traits and other neurodevelopmental traits at each timepoint for each informant. Polygenic scores for ADHD were derived for each child and used to predict ADHD impact. Analyses controlled for child's age at completion of ADHD measures. We also stratified analyses by sex and tested for interactions with sex.

**Results:**

ADHD traits were associated with ADHD impact across informants, ages, and sex (*β* = 0.46–0.64). There were stronger associations among males according to parents, but no sex differences according to teachers. In multivariable analyses, ADHD traits had the strongest association with impact, autistic traits and reading ability predicted parent‐rated impact and pragmatic communication predicted teacher‐rated impact. There was no evidence of an association between genetic risk for ADHD and ADHD impact when controlling for ADHD traits.

**Conclusions:**

ADHD and other neurodevelopmental traits were associated with ADHD impact in children from the general population. This reinforces the importance of inclusive environments for neurodivergent people. Clinicians and educators should consider the presence of impact and multiple neurodevelopmental difficulties when making decisions about support for all children.

## Graphical abstract

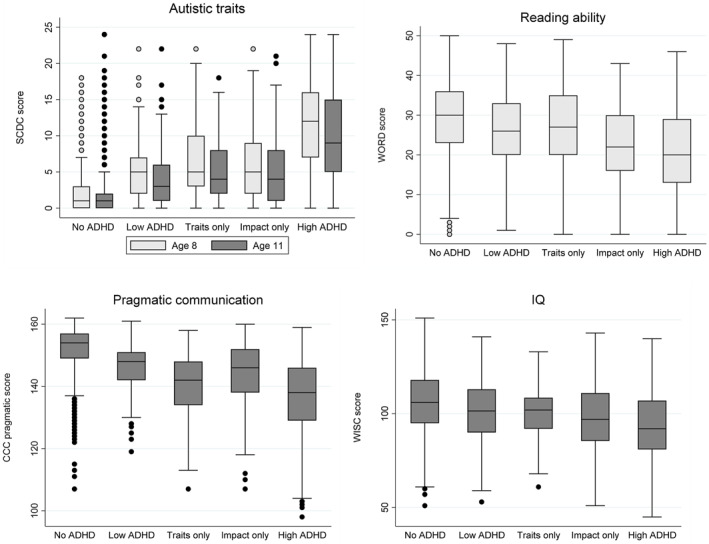

**Key points**

**What's known**

- People diagnosed with ADHD and co‐occurring conditions experience impact on functioning. ADHD traits and other neurodevelopmental traits are distributed continuously in the population and may also lead to impact.

**What's new**

- ADHD traits were associated with ADHD impact in children in the general population, but a substantial amount of variance remained unexplained. Certain neurodevelopmental traits (especially social and communication traits) contributed to ADHD impact even when controlling for ADHD traits.
- There was no evidence of an association between genetic risk for ADHD and impact when controlling for ADHD traits.

**What's relevant**

- This study indicates that clinicians and educators should consider the presence of multiple neurodevelopmental difficulties when making decisions about support and reinforces the importance of inclusive environments for neurodivergent people regardless of diagnoses.

## INTRODUCTION

Attention Deficit Hyperactivity Disorder (ADHD) is a highly heritable and heterogeneous neurodevelopmental condition characterised by symptoms of inattention, hyperactivity, and impulsivity. ADHD is often defined as a categorical diagnosis that requires a minimum number of symptoms, pervasiveness across settings, and impact on functioning. However, ADHD traits are also distributed continuously in the general population (Salum et al., 2014; Stergiakouli et al., 2015), where their association with functional impact is less clear. Impact on functioning, also referred to as impairment in the literature, can be defined and operationalised in different ways (Barkley et al., 2006; Rapee et al., 2012; Üstün & Kennedy, 2009). In this paper, we use the definition from the DSM‐5 criteria for ADHD diagnosis, in which symptoms must have a direct negative impact on social, academic, or occupational functioning (American Psychiatric Association, 2013).

Previous studies have shown a clear positive association between ADHD symptoms and impact on functioning in clinical samples (Barkley et al., 2006; Gadow et al., 2013). The strength of this association can vary depending on several factors, such as the type of measure used, informant, symptom domain, age, and gender. One study of children and adolescents investigated associations between ADHD symptoms and impact stratified by these different factors (Gadow et al., 2013). Teacher‐rated inattentive symptoms in younger females showed the strongest association with impact, while parent‐rated hyperactivity/impulsivity symptoms in younger females showed the weakest association. Therefore, there may be heterogeneity in the impact experienced by children with ADHD, especially in different settings.

Given that ADHD diagnosis is the extreme end of a continuous distribution of traits, it is also useful to understand the association between ADHD traits and impact on functioning in non‐clinical samples. Genetic studies using general population samples show similar risk factors for a clinical diagnosis of ADHD and population‐based ADHD traits (Martin et al., 2014; Stergiakouli et al., 2017). In terms of impact, Gordon et al. (2006) found stronger correlations between ADHD symptoms and impact when combining cases and controls than for cases only. In fact, the relationship between ADHD symptoms and impact appears to be linear, with no evidence for a discrete symptom threshold that would indicate impact (Arildskov et al., 2021). This is consistent with findings about subthreshold ADHD, in which children with some symptoms who do not meet the criteria for a diagnosis still experience impact (Kirova et al., 2019). These findings suggest that ADHD traits are likely to be associated with impact regardless of clinical status.

Another important factor when considering impact on children with ADHD is the presence of co‐occurring conditions. ADHD often co‐occurs with other conditions, and functioning tends to decline as the number of co‐occurring conditions increases (Larson et al., 2011). Previous studies have examined the role of oppositional, conduct and emotional symptoms in ADHD impact (Mörstedt et al., 2015; Ros & Graziano, 2018; Zoromski et al., 2021). In addition, children with co‐occurring ADHD and autism tend to have poorer adaptive and social functioning than children with ADHD only (Rosello et al., 2022). Like ADHD, other neurodevelopmental traits lie on a continuum in the population (Norbury et al., 2004; Stergiakouli et al., 2017), but few studies have examined their contribution to ADHD impact. One study found that children with ADHD were more likely to have pragmatic communication difficulties and that these difficulties partially explained social impact (Staikova et al., 2013). Hence, children with multiple difficulties but who do not necessarily meet diagnostic criteria for a specific neurodevelopmental condition may experience impact without getting appropriate support. The current study was motivated by a need for more population‐based research exploring the impact of ADHD and multiple neurodevelopmental traits. Understanding more about the relationship between neurodevelopmental traits and ADHD impact (especially considering different traits individually and together) will be beneficial to help the assessment of ADHD, to identify those who may be at risk of increased impact and highlight those who may need additional support. It is especially relevant to investigate how this may differ between settings, by age and sex using both continuous and categorical definitions.

In addition, genetic factors are important for ADHD given that it is highly heritable. Polygenic risk scores (PGS) indicating genetic risk for ADHD have been shown to be associated not only with diagnosis but also with ADHD traits in the population (Martin et al., 2014; Stergiakouli et al., 2017). Investigating the relationship between PGS and ADHD impact can shed light on what PGS is capturing and therefore help our understanding of genetic measures and aetiology in future studies.

The overall aim of the present study was to examine the relationship between ADHD traits and co‐occurring neurodevelopmental traits and impact on children in the general population. The first aim was to examine the association between ADHD traits and ADHD‐related impact using a dimensional approach. We expected an association, and explored whether this association would differ by informant, child age and sex. The second aim was to examine the contribution of co‐occurring neurodevelopmental traits (reading skills, cognitive ability, autistic traits, and pragmatic communication) to ADHD impact, using both dimensional and categorical approaches to define ADHD. The dimensional approach focused on continuous measures of traits and impact, whereas the categorical approach involved grouping children, depending on their number of ADHD traits and severity of impact (see Method for details). Whilst dimensional approaches tell us about the patterns of data, categories allow us to compare between individuals who may present with different profiles, which is useful when considering interventions and support. We anticipated that continuous measures of neurodevelopmental traits would be associated with greater impact and expected a dose‐response relationship in categorical groups, in which the group with high ADHD traits and impact would have the most neurodevelopmental difficulties. The third aim was to examine the association between genetic risk for ADHD and ADHD impact using polygenic scores (PGS). As this has not been previously studied, we did not propose any specific hypotheses. To account for other factors associated with impact, we used two different timepoints and both parent and teacher reports of ADHD. We also examined sex differences in these associations.

## MATERIALS AND METHODS

### Sample

This study used data from the Avon Longitudinal Study of Parents and Children (ALSPAC). ALSPAC is an ongoing prospective longitudinal study conducted in the Avon region in the UK. Initially, 14,541 pregnant women with expected delivery dates between 1st April 1991 and 31st December 1992 were recruited to participate, resulting in a sample size of 13,988 children alive at 1 year of age. There was further recruitment of families who met the original criteria over the years, starting when the oldest children were approximately 7 years old. As a result, the total sample size for analysis was 14,901 children alive at 1 year of age.

More details about the methodology and sample used in ALSPAC can be found elsewhere (Boyd et al., 2013; Fraser et al., 2013). Please note that the ALSPAC study website contains details of all the data that is available through a fully searchable data dictionary and variable search tool: http://www.bristol.ac.uk/alspac/researchers/our‐data/.

### Measures

#### ADHD

ADHD traits and ADHD impact were assessed via parent and teacher questionnaires at the approximate ages of 8 and 11 years using the Development and Wellbeing Assessment (DAWBA; Goodman et al., 2000). The DAWBA contains 18 items that correspond to the Diagnostic and Statistical Manual of Mental Disorders (DSM‐IV/DSM‐5) criteria for ADHD diagnosis (American Psychiatric Association, 2013) and additional questions about impact related to ADHD. Supporting Information S1: Figure S1 displays the timeline of when study measures were collected.

For ADHD traits, parents were asked about their child's behaviour compared to other children in the last 6 months, with responses on a 3‐point scale that indicated the severity of the behaviour (no, a little more than others, a lot more than others). Teachers were asked about the child's behaviour over the last school year on a 3‐point scale with different wording (not true, somewhat true, certainly true). Both rating scales were coded 0 to 2, and items were summed into an ADHD rating score using mean imputation for those with no more than 2 missing items. Higher scores indicate more ADHD traits (range 0–36). The teacher DAWBA contained 19 items instead of 18 because the first item was split into two questions (‘makes careless mistakes’ and ‘fails to pay attention’). To keep the traits consistent between parent and teacher reports and to match the DSM symptoms, these two items were combined into a single item by using the highest score of either item. Therefore, both parent‐ and teacher‐reported ADHD traits had the same range.

ADHD impact was assessed in the presence of at least one ADHD trait, that is, when parents answered ‘a lot more than others’ or teachers answered ‘certainly true’ about any ADHD item. In the parent questionnaires, impact was also assessed when parents reported a teacher complaint related to ADHD. In other words, parents who answered that a teacher complained ‘a lot’ about at least one of the main ADHD symptom types (attention, hyperactivity, impulsivity) in the last 6 months were also asked to complete the impact questions.

ADHD impact was defined as the impact items from the DAWBA that matched the impact supplement from the Strengths and Difficulties Questionnaire (SDQ; Goodman, 1999), with five items in the parent report and three items in the teacher report. The parent version included a question about distress (how much the difficulties upset the child) and four questions about functional impact (how the difficulties interfered with day‐to‐day life in four domains: making and keeping friends, learning or schoolwork, getting on with family, and leisure activities). The teacher version included questions about distress, relationship with peers, and classroom learning. Questions were rated on a 4‐point scale (coded 0 to 3) with slightly different wording for parents (not at all, a little, a medium amount, a great deal) and teachers (not at all, only a little, quite a lot, a great deal). Items were summed into an impact rating for those with no more than one missing item. Mean imputation was used for those with a single missing item. Higher ratings indicate greater impact, but the range was different for parents (0–15) and teachers (0–9) due to the different numbers of items.

#### Neurodevelopmental traits

Neurodevelopmental traits (autistic traits, reading ability, cognitive ability, and pragmatic communication) were assessed between ages 7 and 11 **(**Supporting Information S1: Figure S1**).**

Reading ability was assessed face‐to‐face at age 7, using the reading subtest of the Wechsler Objective Reading Dimensions (WORD; Rust et al., 1993). Higher scores indicate more correct items and therefore better reading ability (range 0–50).

Full‐scale IQ was assessed face‐to‐face at age 8, using the Wechsler Intelligence Scale for Children UK (WISC‐III; Wechsler et al., 1992), with a range of 45–151 in this sample.

Autistic traits were assessed via parent questionnaires at the same time as ADHD traits (ages 8 and 11), using the Social and Communication Disorders Checklist (SCDC; Skuse et al., 2005). The SCDC is a reliable and valid measure that has shown accuracy in discriminating autistic individuals from controls, but it does not include any items about special interests or stereotyped motor behaviours (Skuse et al., 2005). Pragmatic communication was assessed via parent questionnaires at age 10, using the pragmatic composite of the Children's Communication Checklist (CCC; Bishop, 1998). Both the SCDC and CCC were rated on a 3‐point scale coded 0 to 2. Higher scores on the SCDC indicate more autistic traits (range 0–24), while higher scores on the CCC indicate better pragmatic communication ability (range 96–162).

#### Other characteristics

Information was available about the children's sex at birth and their age (months) when data were collected. Mothers were asked about their home ownership status when they enrolled into the study and about their educational qualifications in a questionnaire at 32 weeks gestation.

#### Polygenic risk scores

Genetic liability for ADHD was operationalised as PGS for ADHD.

DNA samples were collected from cord blood at birth and were genotyped and imputed as previously described (Dennison et al., 2023). As part of quality control, single nucleotide polymorphisms were filtered based on the Hardy–Weinberg equilibrium *p* < 1 × 10^−4^, genotyping rate <0.95, and minor allele frequency <0.01, and regions of the genome with long range linkage disequilibrium were pruned. Principal components related to ancestry were generated using Plink v1.9.

PGS were based on summary data from the largest available genome‐wide association studies (GWAS) meta‐analysis of ADHD (38,691 cases; 186,843 controls), in which cohorts are mostly of European ancestry (Demontis et al., 2023). PGS for ADHD were derived for each child of the target sample using PRS‐CS (auto) (Ge et al., 2019) and then standardized using Z score transformations.

### Categorical groups

For the categorical approach, only parent data were used since parent reports are the most commonly used in ADHD assessments. Five groups were formed for each timepoint. The first group contained participants with no ADHD traits and therefore no impact data, which were the majority of children in this general population sample. Children with at least one ADHD trait were divided into four groups based on the number of ADHD traits and level of impact. Children with one to five ADHD traits were classed as ‘low ADHD’ when they had no/mild impact and ‘impact only’ when they had moderate/severe impact. Children with six traits or more were classed as ‘traits only’ when they had no/mild impact and ‘high ADHD’ when they had moderate/severe impact.

We used broad definitions of traits and impact because we were interested in understanding more about children in the general population who may not necessarily meet the diagnostic criteria for ADHD. Therefore, we defined moderate/severe impact as answers of ‘quite a lot’ or ‘a great deal’ for any impact domain and high traits as six traits across the symptom domains (inattention or hyperactivity/impulsivity). The DSM‐5 diagnostic criteria are stricter, requiring at least six symptoms from one domain for a diagnosis (American Psychiatric Association, 2013).

### Data analysis

Stata version 17 was used for all analyses.

For the continuous approach, univariable and multivariable linear regressions were conducted with ADHD impact as the outcome. These were separate cross‐sectional analyses for each informant (parent and teacher) and each age (8 and 11). Predictors in the univariable analyses were ADHD traits and each symptom domain (aim 1) and each neurodevelopmental trait (aim 2). Measures of neurodevelopmental traits were continuous and only included in the analysis if they were collected at the same time or before ADHD data (see Supporting Information S1: Figure S1). Therefore, analyses at age 8 included reading ability and autistic traits as predictors, while analyses at age 11 included cognitive ability, pragmatic communication and a later measure of autistic traits. Multivariable analyses included ADHD traits and all neurodevelopmental traits relevant to that timepoint as predictors.

For the categorical approach, we used multinomial regression to compare the level of neurodevelopmental traits in those with no ADHD to those in the other groups (low, impact only, high). We also compared the ADHD groups using logistic regressions. There were few children with more than 6 traits and low impact (traits only), so this group was included for descriptive purposes only.

Analyses were first conducted on all participants and then stratified by sex. In addition, interactions between ADHD score and sex were analysed.

For aim 3, linear regressions were conducted with ADHD PGS as the predictor and ADHD impact as the outcome with and without adjusting for ADHD traits. The first 10 principal components were included as covariates to account for population ancestry effects.

All analyses in this study included the child's age at completion of ADHD measures as a covariate due to age‐related variability in ADHD traits and impact (Faraone et al., 2015). The variables for age of completion in parental reports were leptokurtic and had a highly positive skew due to most participants having the same age but some being older. This was likely due to late completion of questionnaires. For the regression analyses, binary age variables were created in which children with age >1SD from the mean were put into a different category. At age 11, outliers aged over 147 months (*n* < 5) were removed from analyses. These issues were not apparent in the teacher reports, so no outliers had to be removed and variables for age of completion were kept as continuous.

#### Secondary analyses

Parent and teacher data were not directly compared since the sample of children with impact data from both informants was small, but where differences in the results were apparent, we conducted secondary analyses using equivalent impact measures for both informants (parent impact rating without family and leisure items).

#### Sensitivity analyses

Sensitivity analyses for the first aim were conducted in a subsample of children who met diagnostic criteria for ADHD according to parent report at each age. Diagnosis was defined to match DSM‐5 criteria of number of symptoms, impact on functioning and pervasiveness (American Psychiatric Association, 2013). Therefore, children were considered as having a diagnosis if they had six or more ADHD symptoms in at least one domain, moderate or severe parent‐reported impact, and a severe teacher complaint about at least one of the symptom domains.

##### Missing data

The impact section was only completed for children with at least one ADHD trait or parent‐reported teacher complaint at the time of answering. Therefore, missing data were examined separately for parents and teachers and at each age (Supporting Information S1: Table S1). After examining patterns of missing data, we conducted a complete case analysis and compared the results to sensitivity analyses using two other methods of handling missing data (see supplementary text in Supporting Information S1). The other methods used were multiple imputation and assuming a value of zero for children with no impact data when the reason for missingness was having skipped the section (Supporting Information S1: Tables S1–S4).

## RESULTS

### Sample description

The total sample for this study was 12,439 children (49% female), who had data on ADHD available from a parent or teacher for at least one timepoint of interest (age 8 or age 11). If a set of twins met the inclusion criteria, only the first‐born was included. A flowchart for inclusion of study participants is available (Supporting Information S1: Figure S2). The sample size for each category used in the categorical approach can be found in Supporting Information S1: Table S5.

ADHD traits correlated moderately with most functional impact items (0.38–0.60), but weakly with distress (0.17–0.35) (Supporting Information S1: Table S6).

Table 1 shows the demographic characteristics of children with and without ADHD traits according to parent reports. For teacher reports, see Supporting Information S1: Table S7. There were more males with ADHD traits at all timepoints (64%–73%). This pattern was similar when using data from either informant.

**TABLE 1:** Demographics for children with and without Attention Deficit Hyperactivity Disorder (ADHD) traits in parent reports.

	Parent age 8		Parent age 11	
	No ADHD traits	Any ADHD traits	No ADHD traits	Any ADHD traits
Demographics *n* (%)				
Female	3477 (51.1)	453 (36.0)	3376 (51.9)	409 (36.2)
Family owns house^a^	5506 (80.9)	890 (70.8)	5081 (78.1)	816 (72.2)
Mother with A‐levels or higher	2793 (41.0)	455 (36.0)	2615 (40.2)	418 (37.0)
ADHD mean (SD)				
ADHD score (0–36)	2.84 (3.79)	16.38 (8.23)	2.59 (3.66)	16.18 (7.96)
ADHD impact (0–15)	n/a	4.84 (3.50)	n/a	5.18 (3.46)

Abbreviation: ADHD, Attention Deficit Hyperactivity Disorder.

^a^ With or without mortgage.

**FIGURE 1:**
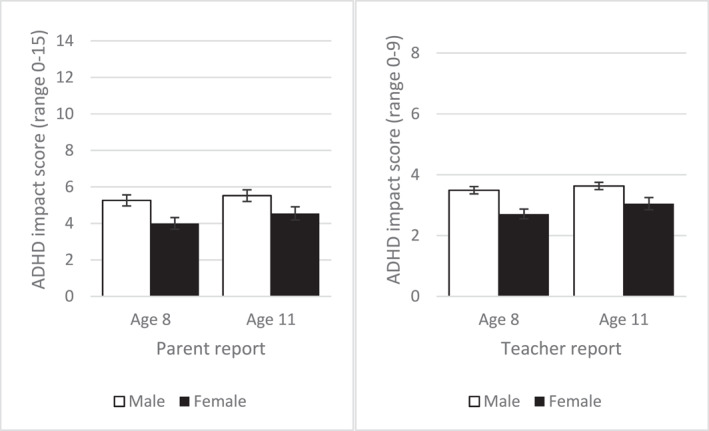
ADHD impact score stratified by sex. Error bars are standard errors of the mean.

Parents and teachers rated males as having significantly more ADHD impact than females at all timepoints (Figure 1; also see Supporting Information S1: Table S8).

### Aim 1—Associations between ADHD traits and ADHD impact

ADHD traits, measured continuously, were associated with both parent and teacher‐reported ADHD impact at ages 8 and 11 (Table 2). Results for each symptom domain (hyperactivity/impulsivity and inattention) are presented in Supporting Information S1: Table S9. Both domains were moderately associated with ADHD impact in all analyses. When including both domains in the regression analysis, inattention was more strongly associated with impact (Age 8: *β* = 0.47, 95%CI = 0.1–0.52; Age 11: *β* = 0.44, 95%CI = 0.38–0.50) than hyperactivity/impulsivity (Age 8: *β* = 0.25, 95%CI = 0.20–0.31; Age11: *β* = 0.28, 95%CI = 0.22–0.34) in parent reports. In teacher reports, associations with impact were similar for both domains.

**TABLE 2:** Results of univariable and multivariable regressions with Attention Deficit Hyperactivity Disorder (ADHD) impact as the outcome and each neurodevelopmental trait as a predictor.

	Univariable		Multivariable	
	*β* (95%CI)	*p*	*β* (95%CI)	*p*
Parent age 8				
ADHD^a^	0.60 (0.55, 0.65)	<0.001	0.40 (0.33, 0.47)	<0.001
Reading ability^b^	−0.20 (−0.27, −0.13)	<0.001	−0.12 (−0.18, −0.06)	<0.001
Autistic traits^a^	0.55 (0.49, 0.60)	<0.001	0.30 (0.23, 0.37)	<0.001
Parent age 11				
ADHD^a^	0.59 (0.54, 0.65)	<0.001	0.40 (0.32, 0.48)	<0.001
IQ^b^	−0.12 (−0.20, −0.04)	0.004	−0.01 (−0.08, 0.06)	0.78
Pragmatic communication^b^	−0.37 (−0.44, −0.30)	<0.001	−0.05 (−0.13, 0.03)	0.22
Autistic traits	0.55 (0.50, 0.61)	<0.001	0.29 (0.20, 0.37)	<0.001
Teacher age 8				
ADHD	0.62 (0.58, 0.65)	<0.001	0.57 (0.51, 0.64)	<0.001
Reading ability	−0.19 (−0.26, −0.12)	<0.001	−0.04 (−0.10, 0.03)	0.235
Autistic traits	0.30 (0.23, 0.37)	<0.001	0.08 (0.01–0.14)	0.025
Teacher age 11				
ADHD	0.56 (0.51, 0.60)	<0.001	0.46 (0.38, 0.54)	<0.001
IQ	−0.26 (−0.33, −0.18)	<0.001	−0.04 (−0.12, 0.04)	0.33
Pragmatic	−0.35 (−0.42, −0.28)	<0.001	−0.22 (−0.32, −0.12)	<0.001
Autistic traits	0.32 (0.25, 0.40)	<0.001	0.08 (−0.02, 0.18)	0.10

*Note*: All analyses include age of completion as a covariate.

Abbreviations: ADHD, Attention Deficit Hyperactivity Disorder; CI, confidence interval.

^a^ Higher scores indicate more traits.

^b^ Higher scores indicate better ability.

Continuously measured ADHD traits were associated with ADHD impact for both males and females across informants and ages (Supporting Information S1: Table S10). There was an interaction between parent‐reported ADHD traits and sex at age 8 (*β* = −0.16) and 11 (*β* = −0.23), with a stronger association for males than females. There was no evidence of interactions with sex in teacher reports. Similarly, inattention and hyperactivity/impulsivity were more strongly associated with impact among males than females according to parents only (Supporting Information S1: Table S10). All sex differences were more marked at age 11 than age 8.

Results did not change when using an equivalent impact measure for both informants (Supporting Information S1: Table S11). Parent‐reported inattention traits were still associated more strongly with impact than hyperactivity/impulsivity and all associations were stronger for males than females.

Associations between continuous ADHD traits and impact were similar for children who met the criteria for a diagnosis of ADHD (Age 8: *β* = 0.38; Age 11: *β* = 0.47) and those who did not (Age 8: *β* = 0.48; Age 11: *β* = 0.48) (Supporting Information S1: Table S12).

### Aim 2—Associations between neurodevelopmental traits and ADHD impact

#### Continuous approach

In the univariable regressions, all neurodevelopmental traits were associated with ADHD impact in the expected direction (Table 2). The associations observed for autistic traits were generally stronger than the associations with other neurodevelopmental traits.

These patterns were still observed when results were stratified by sex, except ADHD impact was not associated with IQ for males (Supporting Information S1: Table S10). Autistic traits had the strongest associations with impact for both sexes according to parents, but associations were stronger for males than females, especially at age 11 (Interaction:*β* = −0.13).

When controlling for all neurodevelopmental traits in a multivariable regression, ADHD score remained the strongest predictor of ADHD impact in all analyses (Table 2). In contrast, IQ was no longer associated with impact. The effect of other traits was small and depended on the informant. Parent‐reported ADHD impact was associated with lower reading ability and more autistic traits, while teacher‐reported impact was associated with lower pragmatic communication scores.

#### Categorical approach

**FIGURE 2:**
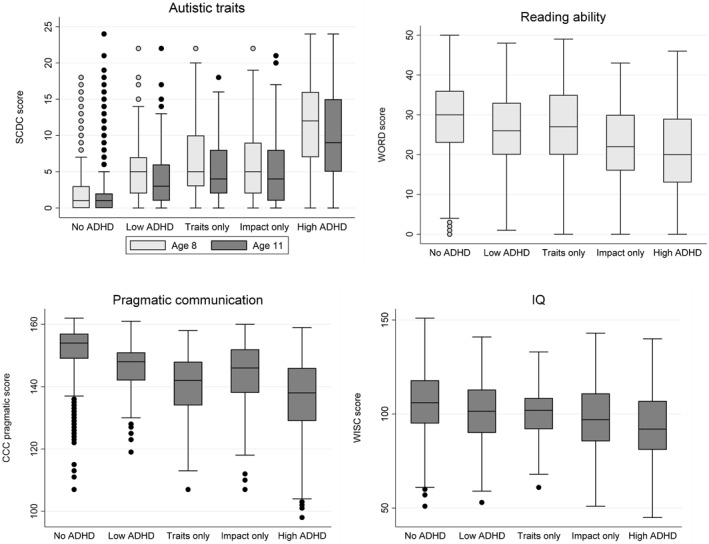
Levels of neurodevelopmental traits for each categorical group. ADHD, Attention Deficit Hyperactivity Disorder; CCC, Children's Communication Checklist; SCDC, Social and Communication Disorders Checklist; WISC, Wechsler Intelligence Scale for Children; WORD, Wechsler Objective Reading Dimensions.

As expected from the way the groups were defined, the ‘high ADHD’ group had the highest ADHD traits and impact scores (Supporting Information S1: Table S5). Mean scores of each neurodevelopmental trait suggested a dose‐response relationship between continuous neurodevelopmental traits and the categorical groups. Children in the ‘high ADHD’ group had the most neurodevelopmental difficulties, those with ‘no ADHD’ had the least, while the other groups fell in the middle (Figure 2).

Results from multivariable multinomial logistic regression analyses using ‘no ADHD’ as the reference group also supported a dose‐response relationship between continuous neurodevelopmental traits and the categorical groups (Table 3). Children in all ADHD groups had more autistic traits and lower ability in reading and pragmatic communication than children with no ADHD traits. IQ was the exception with small effect sizes for all groups and no difference between ‘no ADHD’ and ‘low ADHD’.

**TABLE 3:** Results of a multivariable multinomial logistic regression comparing categorical groups.

	Group	RRR (95% CI)	*p*
Parent age 8			
Reading ability	Low	0.98 (0.96, 0.99)	0.002
	Impact only	0.94 (0.92, 0.95)	<0.001
	High	0.93 (0.91, 0.95)	<0.001
Autistic traits	Low	1.31 (1.26, 1.36)	<0.001
	Impact only	1.36 (1.31, 1.40)	<0.001
	High	1.63 (1.56, 1.69)	<0.001
Parent age 11			
IQ	Low	1.00 (0.99, 1.01)	0.43
	Impact only	0.98 (0.97, 0.99)	<0.001
	High	0.98 (0.96, 0.99)	<0.001
Pragmatic communication	Low	0.95 (0.93, 0.97)	<0.001
	Impact only	0.97 (0.95, 0.99)	0.002
	High	0.92 (0.90, 0.94)	<0.001
Autistic traits	Low	1.25 (1.20, 1.31)	<0.001
	Impact only	1.32 (1.27, 1.37)	<0.001
	High	1.45 (1.39, 1.52)	<0.001

Abbreviations: CI, confidence interval; RRR, relative risk ratio.

Results stratified by sex showed a similar pattern (Supporting Information S1: Table S13). Some effects were only observed for males or females depending on the trait, but confidence intervals overlapped so this was likely due to smaller sample sizes in stratified groups.

When comparing the ADHD groups to each other using logistic regression analyses, children in the ‘high ADHD’ group were more likely to have autistic traits than those in the ‘low ADHD’ (Age 8: OR = 1.29; Age 11: OR = 1.18) and ‘impact only’ groups (Age 8: OR = 1.20; Age11: OR = 1.11) (Supporting Information S1: Table S14). There was also a tendency for children with ‘impact only’ to have more autistic traits than children in the ‘low ADHD’ group (Age 8: OR = 1.06; Age 11:1.08). Regarding other traits, children in the ‘high ADHD’ and ‘impact only’ groups were generally more likely to have neurodevelopmental difficulties than those in the ‘low ADHD’ group, but effect sizes were quite small, with no difference in pragmatic communication between the ‘low’ and ‘impact only’ groups.

### Aim 3—Associations between ADHD PGS and ADHD impact

ADHD PGS had a very weak association with parent‐reported ADHD impact at age 11 (*β* = 0.16) and teacher‐reported impact at both ages (Age 8:*β* = 0.10; Age 11:*β* = 0.09), but not with parent‐reported impact at age 8 (Supporting Information S1: Table S15). When controlling for ADHD traits, there was no evidence of an association between ADHD PGS and ADHD impact in any analysis.

## DISCUSSION

This study examined the association between ADHD and other neurodevelopmental traits and ADHD impact on children in the general population. Results showed that ADHD traits and other neurodevelopmental traits were associated with ADHD impact regardless of sex and age. When controlling for ADHD traits, there was no evidence of an association between ADHD PGS and ADHD impact.

In this study, ADHD traits were associated with ADHD impact across informants (parents and teachers), ages (8 and 11), and sex. However, a substantial amount of variance remained unexplained, despite using an ADHD‐specific measure of impact and the same informant for traits and impact. Both symptom domains were associated with ADHD impact at all timepoints, but inattention showed a stronger association than hyperactivity/impulsivity when using parent reports. These results add to previous research showing that ADHD symptoms and impact on functioning are associated but distinct constructs, and that this association is also present in non‐clinical samples (Arildskov et al., 2021; Gordon et al., 2006).

We investigated the contribution of co‐occurring neurodevelopmental traits to ADHD impact using continuous and categorical approaches to define ADHD. Results from both approaches showed robust associations between neurodevelopmental traits and ADHD impact. When using a continuous approach, some traits explained additional variance in impact even after controlling for ADHD traits. Although results depended on the informant, social and communication traits appeared to be particularly relevant to impact. Aside from ADHD traits, autistic traits had the strongest association with parent‐rated impact, while pragmatic communication had the strongest association with teacher‐rated impact. This parallels research on clinical samples showing that co‐occurring neurodevelopmental difficulties and conditions in children with ADHD were associated with poorer functioning (Cooper et al., 2014; Rosello et al., 2022; Staikova et al., 2013).

It is also important to consider the presence of multiple neurodevelopmental traits. A previous study found a stepwise decline in functioning as the number of co‐occurring conditions increased (Larson et al., 2011). When using a categorical approach in our study, there was a lot of variability within groups, demonstrating heterogeneity in the presence of neurodevelopmental traits. Yet, all analysed groups with at least one ADHD trait were significantly more likely to have neurodevelopmental difficulties than the group with no ADHD traits. In addition, there appeared to be a dose‐response relationship between neurodevelopmental traits and ADHD groups, so those with more than six symptoms and moderate to severe impact were the most likely to have neurodevelopmental difficulties. This is consistent with previous studies using latent class analysis in which participants in the most severe ADHD classes also had the most impact on functioning and the highest levels of co‐occurring conditions (Frick et al., 2023; Todd et al., 2002; Zablotsky et al., 2018). There is also evidence of a dose‐response relationship between the number of co‐occurring neurodevelopmental conditions and impact at school (Fleming et al., 2020). The tendency of multiple neurodevelopmental traits and impact on functioning to co‐occur has important implications for assessment and treatment decisions.

ADHD and co‐occurring neurodevelopmental traits were associated with ADHD impact for both males and females across informants and ages. However, the association between ADHD traits and impact was stronger for males than females when using parent reports. A previous study using a clinical sample did not find sex differences in associations between ADHD symptoms and impact when using parent ratings (Gadow et al., 2013). However, in a different population‐based study, there were sex differences in the way parents rated children according to diagnostic status (Mowlem et al., 2019). In that study, parents rated males meeting ADHD diagnostic criteria as experiencing more impact than males with 5 or more symptoms who did not meet the criteria. In contrast, they did not report differences in impact between females meeting criteria or not. Therefore, it is possible that parents are less able to perceive impact in females than males with a high number of ADHD traits, but further research is necessary.

Both parents and teachers rated males as having more ADHD impact than females, but the interaction between ADHD traits and sex was only observed when using parent ratings. The reason for this discrepancy is not clear. One possibility is that teachers are more likely than parents to attribute impact to ADHD traits in females. Another possibility is that ADHD traits are more strongly associated with impact in the school environment than the home environment for females. In a clinical sample, inattention symptoms were found to be more strongly associated with impact in females than males according to teachers (Gadow et al., 2013). We did not replicate this finding, but together these results suggest that sex differences in the association between ADHD and impact may be context dependent. It is important to note that since we used ADHD data from questionnaires only, it was not possible to determine whether there were true sex differences, or whether these were due to reporter bias. Parents and teachers have been found to underrate ADHD traits and impact in females when using questionnaires compared to direct observations and interviews (Meyer et al., 2020; Mowlem et al., 2019).

In general, associations between ADHD traits and ADHD impact were similar when using parent or teacher reports in our study. However, there were some notable differences. Apart from the sex differences noted above, associations with specific symptom domains showed different patterns. Inattention was more strongly associated with impact than hyperactivity/impulsivity when using parent reports. In addition, different neurodevelopmental traits were associated with ADHD impact according to parents and teachers. Measures of impact were based on the informants' perception, which means ratings may be context specific. Neurodevelopmental traits and their impact can present differently across settings so that each informant provides a unique perspective (Dirks et al., 2012; Kofler et al., 2016). There may also be differences between informants' and children's perceptions of impact, especially in social and emotional functioning (Eiser & Morse, 2001). Therefore, even though it is common in child research to use parent or teacher ratings, it is important for future research to explore the child's perspective of their own ADHD impact.

ADHD is a highly heritable condition, and genetic risk for ADHD is associated with both categorical diagnoses and ADHD traits in the population (Stergiakouli et al., 2015). In our study, ADHD PGS were associated with ADHD impact, but the association was explained by ADHD traits. The ADHD PGS in our study were based on a GWAS of clinically diagnosed ADHD, which captures an ADHD phenotype that includes not only ADHD symptoms but also other clinical features (e.g., impact, pervasiveness). Yet, we did not find evidence of an association between ADHD PGS and impact once we accounted for ADHD traits. Although more genetic studies are needed to clarify these findings and establish the genetic basis of other clinical features related to ADHD (e.g., age at onset), our results lend further support for considering the role of contextual factors in ADHD impact, especially modifiable factors that may help improve functioning.

Associations between ADHD traits and impact were similar at ages 8 and 11. This is consistent with results from a clinical sample in which there were no differences in associations between ADHD symptoms and impact for younger (6–12 years) and older (13–18 years) youth (Gadow et al., 2013). Given that childhood ADHD is associated with negative outcomes in adulthood (Erskine et al., 2016), a useful next step would be to understand the relevance of ADHD traits and their impact on long‐term outcomes.

Overall, our findings suggest that ADHD traits, ADHD impact and other neurodevelopmental traits tend to occur together in the general population, in a dose‐response manner. Previous research showed that ADHD and other neurodevelopmental traits are continuously distributed in the population (Stergiakouli et al., 2015, 2017) and that children with some ADHD symptoms who do not meet the criteria for diagnosis experience impact on their functioning (Kirova et al., 2019). Our results build on these findings and have important implications for research and clinical practice. Children with multiple neurodevelopmental traits associated with different conditions may not necessarily meet the criteria for any diagnosis but still experience impact on their functioning. This was shown in both dimensional and categorical analyses in the current study. It would be useful for schools and clinicians to consider the presence of multiple neurodevelopmental traits in children experiencing impact. Although diagnoses or medication may not be appropriate in these cases (Kazda et al., 2021), these children could still benefit from support at school and at home. Thus, these general population findings add to the growing recognition that more focus is needed on accepting and supporting neurodivergent people, beyond a focus on strict diagnostic thresholds (Sonuga‐Barke et al., 2022).

Our study had several strengths, including a large number of participants, multiple informants and timepoints, dimensional and categorical measures of ADHD, and genetic data. There were also limitations. Due to the cross‐sectional nature of the analyses, we cannot infer causality. As is usual in birth cohorts, attrition was an issue. In particular, ADHD and genetic risk for ADHD have been found to be associated with attrition in ALSPAC (Taylor et al., 2018; Wolke et al., 2009). Therefore, children with high ADHD traits and potentially more impact may be underrepresented in this sample. However, this is unlikely to have affected results since we found similar associations for children who met the criteria for a diagnosis of ADHD and those who did not. Due to the skip rule for the impact section, some children also had missing impact data unrelated to attrition. Nonetheless, results were similar when using different methods for handling missingness in impact data (see supplementary text in Supporting Information S1 and Table S16).

## CONCLUSION

Our results highlight the importance of considering impact on functioning in children with ADHD traits. ADHD traits and other neurodevelopmental traits were associated with ADHD impact regardless of sex and age in this general population sample. In addition, ADHD and other neurodevelopmental difficulties often occur together and may lead to impact even in children who do not meet the criteria for any diagnosis. Clinicians and schools should consider the presence of multiple neurodevelopmental difficulties when making decisions about support and adjustments for children experiencing impact on their functioning.

## AUTHOR CONTRIBUTIONS

**Louise Horstmann:** Conceptualization; data curation; formal analysis; methodology; project administration; software; validation; visualization; writing—original draft; writing—review and editing. **Charlotte A. Dennison:** Methodology; software; writing—review and editing. **Evie Stergiakouli:** Conceptualization; project administration; supervision; writing—review and editing. **Kate Langley:** Conceptualization; funding acquisition; project administration; supervision; writing—review and editing. **Joanna Martin:** Conceptualization; funding acquisition; project administration; software; supervision; writing—review and editing.

## CONFLICT OF INTEREST STATEMENT

JM is an associated editor at Journal of Child Psychology and Psychiatry Advances. KL has received a speaker's fee from Medice on a topic unrelated to this research. All other authors report no conflict of interest.

## ETHICAL CONSIDERATIONS

Ethical approval for the study was obtained from the ALSPAC Ethics and Law Committee and the Local Research Ethics Committees. Informed consent for the use of data collected via questionnaires and clinics was obtained from participants following the recommendations of the ALSPAC Ethics and Law Committee at the time.

## Supporting information

Supporting Information S1

## Data Availability

Data from the ALSPAC participants cannot be made freely available through any third‐party maintained public repository due to the informed consent restrictions. However, data used for this submission can be made available on request to the ALSPAC Executive. The ALSPAC data management plan describes in detail the policy regarding data sharing, which is through a system of managed open access. Full instructions for applying for data access can be found here: http://www.bristol.ac.uk/alspac/researchers/access/. The ALSPAC study website contains details of all the data that are available (http://www.bristol.ac.uk/alspac/researchers/our‐data/).
