# Supplementary material for: Attention Deficit Hyperactivity Disorder and other neurodevelopmental traits are associated with impact on functioning among children in the general population
Source: JCPP Adv. 2025 Mar 7;5(4):e70004. doi: 10.1002/jcv2.70004 (PMC12698278; doi:10.1002/jcv2.70004)
Supplement: Supplementary file 1 — Supporting Information S1 [file JCV2-5-e70004-s001.docx]

# Supporting information


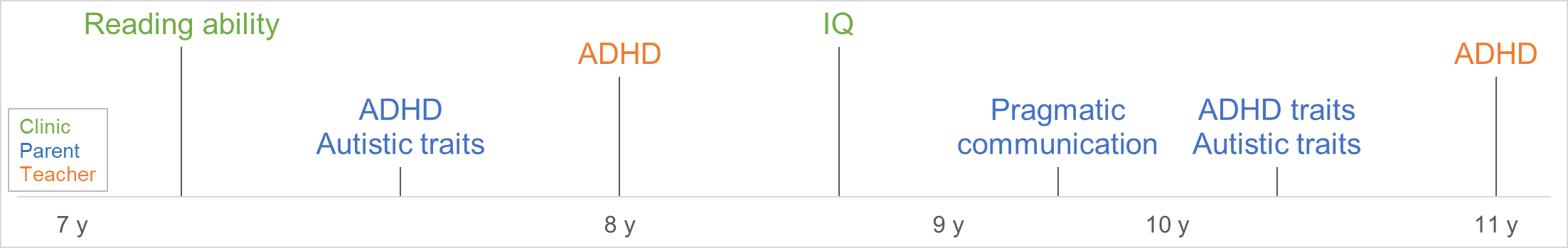


Figure S1. Timeline of study measures in ALSPAC. ADHD: Attention Deficit Hyperactivity Disorder.

# Supplementary text

**Missing data**

In parent reports, ADHD impact had the most missing data (**Table S1)**. There was also a substantial amount of missingness in neurodevelopmental measures that were collected at a different timepoint than ADHD (reading ability, cognitive ability, and pragmatic communication) mostly due to participants having no data at that timepoint. In teacher reports, neurodevelopmental traits had the most missing data as these were based on parent questionnaires and face-to-face appointments at different timepoints.

Almost all missingness in ADHD impact was due to informants having skipped the impact section when they were supposed to answer it. Due to the nature of the skip rule, it is likely that informants skipped the section when children had no impact. This would mean that impact missingness would have been caused by unobserved values of impact and data may be missing not at random (MNAR, Rubin, 1976). In order to better understand this issue, we compared participants with and without impact data in variables that had the strongest correlations with ADHD impact at each timepoint (**Table S2**). Those with missing impact data had lower scores in all correlated measures.

Differences between participants with and without complete data suggest that data were not missing completely at random (MCAR). Although ADHD impact appeared to be MNAR, it is not possible to establish whether data are missing at random (MAR) or MNAR from observed data (White et al., 2011). In addition, the strongest predictor of ADHD impact missingness was ADHD score. When missingness of the outcome is conditional on the analysis variables, complete case analysis (listwise deletion) can be used, whereas multiple imputation is indicated when data are MAR (Sterne et al., 2009). If the outcome is conditional on unobserved variables, then both complete case analysis and multiple imputation would be biased, and a study-specific sensitivity analysis would be indicated (Madley-Dowd et al., 2019).

When using multiple imputation, strong auxiliary variables that predict missing values and missingness should be used to make the MAR assumption more plausible. Auxiliary variables should also be at least moderately correlated with the imputed variable and should not have missing data themselves (Hardt et al., 2012). Unfortunately, despite the large number of variables in this dataset, it was challenging to identify auxiliary variables that met these requirements. Most variables had missing values and few variables other than the analysis variables correlated with ADHD impact. The large proportion of missing data seen in neurodevelopmental traits in the teacher reports also posed a challenge. Most auxiliary variables were collected via parent questionnaires and face-to-face appointments which meant they also had a substantial number of missing values for the sample with teacher-reported ADHD.

Due to these issues, we decided that the best option was to conduct the main analyses using complete case analysis but compare the main results of aim 1 and aim 2 with results using multiple imputation with auxiliary variables and a sensitivity analysis in which missing impact was set to zero when the reason for missingness was skipped section.

For the categorical approach, all children with data at the timepoint of interest were included in the analysis. Some children could not be assigned into any group due to missing data in the ADHD symptom count and/or impact binary variables. There were 508 (6.2%) children with missing data at age 8 and 437 (5.7%) at age 11.

**Multiple Imputation**

Multiple imputation is a simulation procedure used to handle missing data in which multiple imputed datasets with estimated values are generated (Sterne et al., 2009). Each imputed dataset is then analysed using standard methods, and their results combined using Rubin’s rules (Rubin, 1976). In this study, multiple imputation by chained equations was conducted separately for each timepoint using the Stata command *mi*.

There was a large proportion of missing data at each timepoint (46-63%). The largest fraction of missing information (FMI) value ranged between 0.38-0.65 depending on the timepoint. As a rule of thumb, the number of imputations should be m ≥100 x FMI (White et al., 2011). Therefore, we generated 200 imputed datasets, which should be sufficient for this study.

Linear regressions were used to impute normal continuous variables and logistic regressions were used to impute binary and categorical variables. For continuous variables with a limited range and slightly skewed distributions, Predictive Mean Matching was used (White et al., 2011).

The imputation models included the analysis variables and auxiliary variables. Auxiliary variables were determined based on linear and logistic regressions with each imputed variable or a binary indicator of their missingness as the outcome.

In parent reports, the auxiliary variables used were parent-reported SDQ total difficulties assessed at a similar age to ADHD and maternal and paternal education at birth (**Table S3**). At age 11, previous ADHD and autistic scores were also used. No auxiliary variables were used for variables with very few missing values (**Table S1**).

In teacher reports, the auxiliary variables used were teacher-reported SDQ total difficulties and SDQ impact score assessed at the same age as ADHD, a binary indicator of prematurity, maternal and paternal education at birth and maternal depression, which was measured by the Edinburgh post-natal depression score approximately 8 weeks after birth (**Table S4**). Age and ADHD score had very few or no missing values and did not require auxiliary variables (**Table S1**).

**Sensitivity Analyses**

**Table S16** shows a comparison of results for aim 1 and aim 2 using complete case analysis, multiple imputation, and missing impact set to zero for those who skipped the impact section. Results correspond to the beta coefficient of ADHD score in univariable and multivariable linear regressions with ADHD impact as the outcome.

The results were similar regardless of the method used to handle missing data. Complete case analysis and multiple imputation resulted in virtually the same results. Assuming that all children had an ADHD impact score of zero when their parent or teacher skipped the impact section is an extreme case scenario and resulted in slightly larger effect sizes. Notwithstanding, confidence intervals still overlapped with the results from the complete case analysis and multiple imputation.

Table S1. Reasons for missingness and proportion of missing data for each variable.

|  | **Reasons for missingness** | **Parent**  **Age 8**  **(n = 1257)** | **Parent**  **Age 11**  **(n = 1131)** | **Teacher**  **Age 8**  **(n = 1752)** | **Teacher**  **Age 11**  **(n = 1745)** |
| --- | --- | --- | --- | --- | --- |
|  |  | **N missing (%)** | **N missing (%)** | **N missing (%)** | **N missing (%)** |
| ADHD/HI/IA score | Item non-response | 17 (1.4) | 12 (1.1) | 5 (0.3) | 2 (0.1) |
| ADHD impact | Section skipped  Item non-response | 349 (27.8) | 328 (29) | 170 (9.7) | 227 (13) |
| Autistic traits | Section skipped  Item non-response  No data at timepoint (teacher only) | 16 (1.3) | 11 (1) | 815 (46.5) | 966 (55.4) |
| Reading ability | No data at timepoint  Failure to complete task | 293 (23.3) | n/a | 832 (47.5) | n/a |
| Cognitive ability | No data at timepoint  Failure to complete task | n/a | 320 (28.3) | n/a | 1012 (58) |
| Pragmatic communication | No data at timepoint  Section skipped  Item non-response | n/a | 165 (14.6) | n/a | 941 (53.9) |
| Age at completion | Item non-response | 7 (0.6) | 1 (0.1) | 4 (0.2) | 0 (0) |
| Notes: ADHD: Attention Deficit Hyperactivity Disorder; HI: Hyperactivity-impulsivity; IA: Inattention | | | | | |

Table S2. Comparison between participants with and without impact data on variables that correlate with impact at each timepoint

|  | **Corr impact^1^** | **Missing impact** | | **Complete impact** | | **Comparison** |
| --- | --- | --- | --- | --- | --- | --- |
|  | **r** | **n** | **Mean (SD)** | **n** | **Mean (SD)** | **Test statistic** |
| **Parent Age 8** |  |  |  |  |  |  |
| ADHD traits | 0.60 | 343 | 11.43 (5.87) | 897 | 18.28 (8.23) | *t*(1238) = 14.10, *p* <.0001 |
| Autistic traits | 0.55 | 341 | 5.27 (4.42) | 900 | 7.90 (5.83) | *t*(1239) = 7.54, *p* <.0001 |
| SDQ difficulties | 0.50 | 299 | 10.79 (5.60) | 755 | 13.72 (5.79) | *t*(1052) = 7.46, *p* <.0001 |
| **Parent Age 11** |  |  |  |  |  |  |
| ADHD traits | 0.59 | 324 | 12.31 (6.01) | 795 | 17.76 (8.13) | *t*(1117) = 10.92, *p* <.0001 |
| Autistic traits | 0.55 | 320 | 5.09 (4.39) | 800 | 7.33 (5.93) | *t*(1118) = 6.12, *p* <.0001 |
| SDQ difficulties | 0.51 | 259 | 10.20 (5.41) | 646 | 12.56 (5.89) | *t*(903) = 5.59, *p* <.0001 |
| **Teacher Age 8** |  |  |  |  |  |  |
| ADHD traits | 0.62 | 169 | 10.68 (5.87) | 1578 | 16.18 (8.49) | *t*(1745) = 8.22, *p* <.0001 |
| SDQ difficulties | 0.70 | 167 | 8.30 (5.40) | 1580 | 12.02 (6.28) | *t*(1745) = 7.36, *p* <.0001 |
| SDQ impact | 0.74 | 155 | 1.56 (2.16) | 1543 | 2.95 (2.49) | *t*(1696) = 6.69, *p* <.0001 |
| **Teacher Age 11** |  |  |  |  |  |  |
| ADHD traits | 0.55 | 226 | 10.61 (5.61) | 1517 | 16.90 (8.29) | *t*(1741) = 11.04, *p* <.0001 |
| SDQ difficulties | 0.69 | 227 | 9.15 (5.35) | 1518 | 13.19 (6.47) | *t*(1743) = 8.94, *p* <.0001 |
| SDQ impact | 0.75 | 216 | 1.61 (2.38) | 1497 | 3.16 (2.67) | *t*(1711) = 8.06, *p* <.0001 |
| Notes: ^1^ Correlation with ADHD impact; SD: standard deviation; ADHD: Attention Deficit Hyperactivity Disorder; SDQ: Strengths and Difficulties Questionnaire | | | | | | |

Table S3. Imputed and auxiliary variables used in the multiple imputation in parent reports

| **Imputed variable** | **Auxiliary variables** |
| --- | --- |
| ADHD impact Age 8 | SDQ (Age 6 and 8), maternal education |
| Reading ability  Pragmatic communication | Maternal and paternal education |
| ADHD impact Age 11 | SDQ (Age 11), ADHD score (Age 8), autistic traits (Age 8) |
| IQ | SDQ (Age 11), ADHD score (Age 8), autistic traits (Age 8), maternal and paternal education |
| Notes: ADHD: Attention Deficit Hyperactivity Disorder; SDQ: Strengths and Difficulties Questionnaire | |

Table S4. Imputed and auxiliary variables used in the multiple imputation in teacher reports

| **Imputed variable** | **Auxiliary variables from teacher reports** | **Auxiliary variables from parent reports** |
| --- | --- | --- |
| ADHD impact Age 8  Autistic traits Age 8 | SDQ total and impact (Age 8) | Prematurity |
| Reading ability | - | Prematurity |
| ADHD impact Age 11 | SDQ total and impact (Age 11) | - |
| Pragmatic communication  Autistic traits Age 11 | SDQ total and impact (Age 11) | Maternal depression |
| IQ | SDQ total and impact (Age 11) | Maternal and paternal education, maternal depression |
| Notes: ADHD: Attention Deficit Hyperactivity Disorder; SDQ: Strengths and Difficulties Questionnaire | | |

ALSPAC sample
n = 15,645

Alive at 1 year of age
n = 14,868

Children with ADHD data
n = 12,587

Excluded second-born in set of siblings n = 148

Study sample
n = 12, 439

Parent Age 8 n = 8,097

Parent Age 11 n = 7,670

Teacher Age 8 n = 6,278

Teacher Age 11 n = 7,558

Figure S2. Flowchart of participants from the Avon Longitudinal Study of Parents and Children. ALSPAC: Avon Longitudinal Study of Parents and Children; ADHD: Attention Deficit Hyperactivity Disorder

Table S5. Descriptive statistics for each categorical group

|  | **No ADHD** | **Low ADHD** | **Traits only** | **Impact only** | **High ADHD** |
| --- | --- | --- | --- | --- | --- |
|  | **Mean (SD)** | **Mean (SD)** | **Mean (SD)** | **Mean (SD)** | **Mean (SD)** |
| **Parent Age 8** | **n = 6700** | **n = 261** | **n = 51** | **n = 278** | **n = 299** |
| ADHD traits (0-36)^1^ | 2.84 (3.79) | 12.04 (4.91) | 23.51 (5.04) | 14.06 (4.51) | 26.84 (5.39) |
| ADHD impact (0-15)^1^ | n/a | 1.54 (1.33) | 2.37 (1.68) | 5.26 (2.50) | 7.75 (3.00) |
| Reading ability (0-52)^2^ | 29.11 (8.82) | 26.53 (9.80) | 26.92 (11.05) | 22.58 (9.71) | 21.02 (10.59) |
| Autistic traits (0-24)^1^ | 2.01 (2.51) | 5.21 (3.82) | 7.20 (5.36) | 6.15 (4.74) | 11.97 (6.08) |
| **Parent Age 11** | **n = 6437** | **n = 207** | **n = 30** | **n = 290** | **n = 269** |
| ADHD traits (0-36) | 2.59 (3.66) | 11.88 (4.72) | 24.50 (5.17) | 13.48 (4.12) | 26.21 (5.93) |
| ADHD impact (0-15) | n/a | 1.78 (1.40) | 2.50 (1.55) | 5.29 (2.30) | 7.92 (3.20) |
| IQ (45-151)^2^ | 106.25 (15.94) | 103.22 (16.75) | 99.18 (17.30) | 99.27 (16.20) | 94.62 (19.16) |
| Pragmatic communication(96-162)^2^ | 152.04 (6.31) | 146.41 (8.74) | 136.96 (12.58) | 146.37 (9.53) | 136.78 (13.40) |
| Autistic traits (0-24) | 1.64 (2.46) | 4.65 (4.01) | 8.31 (5.34) | 5.69 (4.59) | 11.00 (6.53) |
| ADHD: Attention Deficit Hyperactivity Disorder, SD: standard deviation. ^1^ higher scores indicate more traits or impact; ^2^ higher scores indicate better ability. | | | | | |

Table S6. Spearman correlations between ADHD score and each impact item

|  | **Distress** | **School** | **Friends** | **Family** | **Leisure** |
| --- | --- | --- | --- | --- | --- |
| **Parent** |  |  |  |  |  |
| Age 8 | 0.35 | 0.41 | 0.48 | 0.45 | 0.50 |
| Age 11 | 0.28 | 0.38 | 0.50 | 0.42 | 0.45 |
| **Teacher** |  |  |  |  |  |
| Age 8 | 0.17 | 0.60 | 0.57 | - | - |
| Age 11 | 0.20 | 0.55 | 0.50 | - | - |

Table S7. Demographics for children with and without ADHD traits in teacher reports

|  | **Teacher Age 8** | | **Teacher Age 11** | |
| --- | --- | --- | --- | --- |
|  | **No ADHD traits** | **ADHD traits** | **No ADHD traits** | **ADHD traits** |
| **Demographics n(%)** |  |  |  |  |
| Female | 2534 (56) | 577 (32.9) | 3274 (56.3) | 474 (27.2) |
| Family owns house^1^ | 3422 (75.6) | 1142 (65.2) | 4095 (70.5) | 961 (55.1) |
| Mother with A-levels or higher | 1521 (33.6) | 458 (26.1) | 1774 (30.5) | 370 (21.2) |
| **ADHD Mean (SD)** |  |  |  |  |
| ADHD score (0-36) | 2.58 (3.34) | 15.64 (8.43) | 2.24 (3.26) | 16.08 (8.26) |
| ADHD impact (0-9) | n/a | 3.24 (1.96) | n/a | 3.48 (2.06) |
| **Notes:** ADHD: Attention Deficit Hyperactivity Disorder  **^1^** with or without a mortgage | | | | |

Table S8. ADHD impact score stratified by sex

|  | **Male** |  | **Female** |  | **Sex differences** |
| --- | --- | --- | --- | --- | --- |
|  | **N** | **Mean (SD)** | **N** | **Mean (SD)** | **t-test** |
| **Parent** |  |  |  |  |  |
| Age 8 | 604 | 5.26 (3.73) | 304 | 4.00 (2.82) | *t*(906) = 5.18, *p* < .0001 |
| Age 11 | 525 | 5.52 (3.62) | 278 | 4.55 (3.07) | *t*(801) = 3.82, *p* = .0001 |
| **Teacher** |  |  |  |  |  |
| Age 8 | 1076 | 3.49 (1.96) | 506 | 2.71 (1.83) | *t*(1580) = 7.52, *p* < .0001 |
| Age 11 | 1136 | 3.63 (2.07) | 382 | 3.05 (1.95) | *t*(1516) = 4.77, *p* < .0001 |
| Notes: SD: standard deviation | | | | | |

Table S9. Results of univariable and multivariable regressions with ADHD impact as the outcome and each ADHD symptom domain as a predictor

|  | **Univariable** | | **Multivariable** | |
| --- | --- | --- | --- | --- |
|  | **β (95%CI)** | ***p*** | **β (95%CI)** | ***p*** |
| **Parent Age 8** |  |  |  |  |
| HI | 0.44 (0.38, 0.50) | <0.001 | 0.25 (0.20, 0.31) | <0.001 |
| IA | 0.57 (0.52, 0.62) | <0.001 | 0.47 (0.41, 0.52) | <0.001 |
| **Parent Age 11** |  |  |  |  |
| HI | 0.45 (0.39, 0.51) | <0.001 | 0.28 (0.22, 0.34) | <0.001 |
| IA | 0.55 (0.49, 0.60) | <0.001 | 0.44 (0.38, 0.50) | <0.001 |
| **Teacher Age 8** |  |  |  |  |
| HI | 0.53 (0.48, 0.57) | <0.001 | 0.39 (0.34, 0.43) | <0.001 |
| IA | 0.51 (0.46, 0.55) | <0.001 | 0.35 (0.31, 0.39) | <0.001 |
| **Teacher Age 11** |  |  |  |  |
| HI | 0.48 (0.43, 0.52) | <0.001 | 0.36 (0.31, 0.40) | <0.001 |
| IA | 0.45 (0.40, 0.49) | <0.001 | 0.31 (0.26, 0.35) | <0.001 |
| All analyses adjusted for age; CI: confidence interval; HI: hyperactivity/impulsivity; IA: inattention | | | | |

Table S10. Results of univariable regressions with ADHD impact as the outcome stratified by sex

|  | **Male** | | **Female** | | **Interaction** | |
| --- | --- | --- | --- | --- | --- | --- |
|  | **β (95%CI)** | ***p*** | **β (95%CI)** | ***p*** | **β (95%CI)** | ***p*** |
| **Parent Age 8** |  |  |  |  |  |  |
| ADHD | 0.61 (0.55, 0.68) | <0.001 | 0.55 (0.45, 0.65) | <0.001 | -0.16 (-0.29, -0.03) | 0.02 |
| HI | 0.46 (0.39, 0.53) | <0.001 | 0.38 (0.27, 0.48) | <0.001 | -0.13 (-0.25, -0.01) | 0.03 |
| IA | 0.58 (0.52, 0.65) | <0.001 | 0.52 (0.43, 0.62) | <0.001 | -0.17 (-0.29, -0.05) | 0.005 |
| Reading | -0.19 (-0.28, -0.10) | <0.001 | -0.18 (-0.31, -0.05) | <0.001 | 0.02 (-0.19, 0.23) | 0.85 |
| Autistic traits | 0.57 (0.50, 0.63) | <0.001 | 0.45 (0.35, 0.55) | <0.001 | -0.11 (-0.21, -0.02) | 0.02 |
| **Parent Age 11** |  |  |  |  |  |  |
| ADHD | 0.64 (0.57, 0.70) | <0.001 | 0.45 (0.35, 0.56) | <0.001 | -0.23 (-0.37, -0.10) | 0.001 |
| HI | 0.50 (0.43, 0.58) | <0.001 | 0.27 (0.15, 0.38) | <0.001 | -0.19 (-0.29, -0.08) | 0.001 |
| IA | 0.57 (0.50, 0.64) | <0.001 | 0.47 (0.37, 0.58) | <0.001 | -0.22 (-0.37, -0.07) | 0.004 |
| IQ | -0.09 (-0.19, 0.01) | 0.08 | -0.22 (-0.35, -0.08) | 0.002 | -0.32 (-0.78, 0.15) | 0.18 |
| Pragmatic | -0.42 (-0.50, -0.33) | <0.001 | -0.23 (-0.36, -0.11) | <0.001 | 0.06 (0.01, 0.10) | 0.01 |
| Autistic traits | 0.61 (0.54, 0.68) | <0.001 | 0.37 (0.26, 0.48) | <0.001 | -0.13 (-0.21, -0.05) | 0.001 |
| **Teacher Age 8** |  |  |  |  |  |  |
| ADHD | 0.60 (0.55, 0.65) | <0.001 | 0.59 (0.51, 0.66) | <0.001 | 0.03 (-0.05, 0.10) | 0.51 |
| HI | 0.51 (0.45, 0.56) | <0.001 | 0.50 (0.42, 0.58) | <0.001 | 0.05 (0.00, 0.11) | 0.06 |
| IA | 0.49 (0.44, 0.54) | <0.001 | 0.48 (0.40, 0.55) | <0.001 | -0.04 (-0.13, 0.06) | 0.48 |
| Reading | -0.16 (-0.24, -0.08) | <0.001 | -0.22 (0.05, 0.13) | <0.001 | -0.09 (-0.29, 0.11) | 0.39 |
| Autistic traits | 0.31 (0.23, 0.38) | <0.001 | 0.25 (0.13, 0.37) | <0.001 | -0.04 (-0.14, 0.05) | 0.36 |
| **Teacher Age 11** |  |  |  |  |  |  |
| ADHD | 0.55 (0.50, 0.59) | <0.001 | 0.55 (0.46, 0.63) | <0.001 | 0.02 (-0.06, 0.11) | 0.59 |
| HI | 0.47 (0.42, 0.52) | <0.001 | 0.43 (0.34, 0.52) | <0.001 | -0.01 (-0.07, 0.06) | 0.88 |
| IA | 0.43 (0.37, 0.48) | <0.001 | 0.47 (0.38, 0.55) | <0.001 | 0.04 (-0.07, 0.15) | 0.48 |
| IQ | -0.26 (-0.35, -0.18) | <0.001 | -0.26 (-0.42, -0.11) | 0.001 | -0.02 (-0.44, 0.39) | 0.91 |
| Pragmatic | -0.36 (-0.44, -0.28) | <0.001 | -0.34 (-0.48, -0.19) | <0.001 | 0.19 (-0.77, 1.15) | 0.70 |
| Autistic traits | 0.32 (0.23, 0.40) | <0.001 | 0.36 (0.21, 0.51) | <0.001 | 0.03 (-0.08, 0.13) | 0.61 |
| All analyses adjusted for age; CI: confidence interval, ADHD: Attention Deficit Hyperactivity Disorder, HI: hyperactivity/impulsivity, IA: inattention | | | | | | |

Table S11. Results of univariable regressions with ADHD impact as the outcome and ADHD traits as predictors for all children and stratified by sex using an equivalent impact measure for parents as the measure for teachers

|  | **All^1^** |  | **Male^1^** |  | **Female^1^** |  | **Interaction^2^** |  |
| --- | --- | --- | --- | --- | --- | --- | --- | --- |
|  | **β (95%CI)** | ***p*** | **β (95%CI)** | ***p*** | **β (95%CI)** | **p** | **β (95%CI)** | **p** |
| **Parent Age 8** |  |  |  |  |  |  |  |  |
| **ADHD** | 0.55 (0.50, 0.61) | <0.001 | 0.57 (0.51, 0.64) | <0.001 | 0.46 (0.36, 0.56) | <0.001 | -0.18 (-0.32, -0.05) | 0.007 |
| **HI** | 0.37 (0.31, 0.43) | <0.001 | 0.40 (0.33, 0.47) | <0.001 | 0.26 (0.15, 0.37) | <0.001 | -0.17 (-0.29, -0.04) | 0.009 |
| **IA** | 0.56 (0.51, 0.62) | <0.001 | 0.58 (0.51, 0.64) | <0.001 | 0.50 (0.40, 0.60) | <0.001 | -0.17 (-0.29, -0.05) | 0.005 |
| **Parent Age 11** |  |  |  |  |  |  |  |  |
| **ADHD** | 0.53 (0.48, 0.59) | <0.001 | 0.59 (0.52, 0.66) | <0.001 | 0.38 (0.27, 0.49) | <0.001 | -0.21 (-0.35, -0.07) | 0.004 |
| **HI** | 0.36 (0.30, 0.43) | <0.001 | 0.43 (0.36, 0.51) | <0.001 | 0.16 (0.05, 0.28) | 0.007 | -0.19 (-0.30, -0.08) | 0.001 |
| **IA** | 0.55 (0.49, 0.60) | <0.001 | 0.58 (0.51, 0.65) | <0.001 | 0.46 (0.36, 0.57) | <0.001 | -0.18 (-0.33, -0.03) | 0.02 |
| All analyses adjusted for age. CI: confidence interval; ADHD: Attention Deficit Hyperactivity Disorder ; HI : hyperactivity; IA: inattention | | | | | | | | |

Table S12. Results of univariable regressions with ADHD impact as the outcome and ADHD traits as a predictor stratified by meeting criteria for an ADHD diagnosis

|  | **Met criteria for diagnosis** | | | **No diagnosis** | | |
| --- | --- | --- | --- | --- | --- | --- |
|  | **n** | **β (95%CI)** | **p** | **n** | **β (95%CI)** | **p** |
| **Parent Age 8** |  |  |  |  |  |  |
| ADHD | 147 | 0.38 (0.23, 0.54) | <0.001 | 740 | 0.48 (0.42, 0.54) | <0.001 |
| **Parent Age 11** | |  |  |  |  |  |
| ADHD | 130 | 0.47 (0.32, 0.62) | <0.001 | 659 | 0.48 (0.41, 0.54) | <0.001 |
| All analyses adjusted for age. CI: confidence interval; ADHD: Attention Deficit Hyperactivity Disorder; HI: hyperactivity; IA: inattention | | | | | | |

Table S13. Results of a multivariable multinomial logistic regression comparing categorical groups stratified by sex

|  | **Group** | **Male** | | **Female** | |
| --- | --- | --- | --- | --- | --- |
|  |  | **RRR (95% CI)** | ***p*** | **RRR (95% CI)** | ***p*** |
| **Parent Age 8** |  |  |  |  |  |
| **Reading** | Low | 0.99 (0.97, 1.01) | 0.23 | 0.96 (0.93, 0.98) | 0.001 |
|  | Impact only | 0.94 (0.92, 0.96) | <0.001 | 0.94 (0.91, 0.96) | <0.001 |
|  | High | 0.94 (0.92, 0.96) | <0.001 | 0.91 (0.88, 0.94) | <0.001 |
| **Autistic traits** | Low | 1.30 (1.24, 1.36) | <0.001 | 1.32 (1.24, 1.40) | <0.001 |
|  | Impact only | 1.32 (1.26, 1.37) | <0.001 | 1.42 (1.34, 1.51) | <0.001 |
|  | High | 1.59 (1.52, 1.67) | <0.001 | 1.66 (1.54, 1.79) | <0.001 |
| **Parent Age 11** |  |  |  |  |  |
| **IQ** | Low | 0.99 (0.98, 1.00) | 0.20 | 1.00 (0.98, 1.02) | 0.81 |
|  | Impact only | 0.98 (0.97, 0.99) | 0.003 | 0.98 (0.96, 0.99) | 0.005 |
|  | High | 0.98 (0.97, 1.00) | 0.009 | 0.96 (0.94, 0.98) | <0.001 |
| **Pragmatic** | Low | 0.97 (0.94, 1.00) | 0.08 | 0.93 (0.89, 0.96) | <0.001 |
| **communication** | Impact only | 0.97 (0.95, 1.00) | 0.02 | 0.98 (0.94, 1.01) | 0.20 |
|  | High | 0.93 (0.90, 0.95) | <0.001 | 0.92 (0.88, 0.96) | <0.001 |
| **Autistic traits** | Low | 1.26 (1.19, 1.33) | <0.001 | 1.25 (1.16, 1.34) | <0.001 |
|  | Impact only | 1.32 (1.26, 1.39) | <0.001 | 1.33 (1.24, 1.41) | <0.001 |
|  | High | 1.47 (1.39, 1.55) | <0.001 | 1.43 (1.33, 1.55) | <0.001 |
| RRR: relative risk ratio; CI: confidence interval. | | | | | |

Table S14. Results of multivariable logistic regressions comparing ADHD groups

|  | **Low vs High** |  | **Low vs Impact only** |  | **Impact only vs High** |  |
| --- | --- | --- | --- | --- | --- | --- |
|  | **OR (95% CI)** | **p** | **OR (95% CI)** | ***p*** | **OR (95% CI)** | ***p*** |
| **Parent Age 8** | **N = 412** |  | **N = 419** |  | **N = 441** |  |
| **Reading ability^1^** | 0.95 (0.93, 0.98) | <0.001 | 0.96 (0.94, 0.98) | <0.001 | 0.98 (0.96, 1.00) | 0.13 |
| **Autistic traits^2^** | 1.29 (1.22, 1.36) | <0.001 | 1.06 (1.01, 1.11) | 0.02 | 1.20 (1.15, 1.25) | <0.001 |
| **Parent Age 11** | **N = 306** |  | **N = 340** |  | **N = 364** |  |
| **IQ^1^** | 0.98 (0.96-0.99) | 0.002 | 0.98 (0.97-1.00) | 0.02 | 0.99 (0.98-1.00) | 0.14 |
| **Pragmatic communication^1^** | 0.96 (0.93-0.99) | 0.01 | 1.02 (0.99-1.05) | 0.19 | 0.95 (0.92-0.98) | <0.001 |
| **Autistic traits** | 1.18 (1.11-1.25) | <0.001 | 1.08 (1.02-1.15) | 0.01 | 1.11 (1.06-1.17) | <0.001 |
| CI: confidence interval; ^1^ OR<1: lower ability; ^2^ OR>1: more traits | | | | | | |

Table S15 Results of linear regressions with ADHD impact as the outcome and ADHD PRS as a predictor with and without ADHD traits as a covariate

|  | **Adjusted for PCA and age only** | | | **Adjusted for ADHD traits** | | |
| --- | --- | --- | --- | --- | --- | --- |
|  | **n** | **β (95% CI)** | ***p*** | **n** | **β (95% CI)** | ***p*** |
| **Parent Age 8** | 637 | 0.06 (-0.02, 0.13) | 0.15 | 630 | 0.00 (-0.07, 0.06) | 0.88 |
| **Parent Age 11** | 575 | 0.16 (0.08, 0.24) | <0.001 | 571 | 0.05 (-0.02, 0.12) | 0.14 |
| **Teacher Age 8** | 897 | 0.10 (0.03, 0.16) | 0.005 | 894 | 0.03 (-0.03, 0.08) | 0.30 |
| **Teacher Age 11** | 858 | 0.09 (0.03, 0.16) | 0.007 | 858 | 0.04 (-0.01, 0.10) | 0.13 |
| PCA: principal component analysis; ADHD: Attention Deficit Hyperactivity Disorder; CI: confidence interval | | | | | | |

Table S16. Comparison of regression coefficients of ADHD score as a predictor of ADHD impact using different methods for handling missing data

|  | **Univariable** | | **Multivariable** | |
| --- | --- | --- | --- | --- |
|  | **n** | **B (95%CI)** | **n** | **B (95%CI)** |
| **Parent Age 8** |  |  |  |  |
| Complete case analysis | 891 | 0.25 (0.23, 0.28) | 681 | 0.16 (0.13, 0.19) |
| Multiple imputation | 1257 | 0.25 (0.23, 0.27) | 1257 | 0.17 (0.15, 0.20) |
| Missing impact set to 0 | 1229 | 0.28 (0.26, 0.30) | 936 | 0.21 (0.18, 0.24) |
| **Parent Age 11** |  |  |  |  |
| Complete case analysis | 794 | 0.25 (0.23, 0.27) | 518 | 0.16 (0.13, 0.20) |
| Multiple imputation | 1131 | 0.24 (0.22, 0.26) | 1131 | 0.17 (0.14, 0.19) |
| Missing impact set to 0 | 1116 | 0.28 (0.26, 0.30) | 728 | 0.21 (0.18, 0.24) |
| **Teacher Age 8** |  |  |  |  |
| Complete case analysis | 1574 | 0.14 (0.13, 0.15) | 644 | 0.13 (0.11, 0.14) |
| Multiple imputation | 1752 | 0.14 (0.13, 0.15) | 1752 | 0.13 (0.12, 0.14) |
| Missing impact set to 0 | 1740 | 0.15 (0.14, 0.16) | 721 | 0.14 (0.12, 0.16) |
| **Teacher Age 11** |  |  |  |  |
| Complete case analysis | 1517 | 0.14 (0.13, 0.15) | 428 | 0.12 (0.10, 0.14) |
| Multiple imputation | 1745 | 0.14 (0.12, 0.15) | 1745 | 0.12 (0.11, 0.13) |
| Missing impact set to 0 | 1743 | 0.16 (0.15, 0.17) | 512 | 0.13 (0.11, 0.15) |
| Notes: CI: confidence interval | | | | |
